# Percentage Gleason pattern 4 and PI-RADS score predict upgrading in biopsy Grade Group 2 prostate cancer patients without cribriform pattern

**DOI:** 10.1007/s00345-022-04161-6

**Published:** 2022-10-03

**Authors:** Margaretha A. van der Slot, Neslisah Seyrek, Charlotte F. Kweldam, Michael A. den Bakker, Martijn B. Busstra, Melanie Gan, Sjoerd Klaver, John B. W. Rietbergen, Geert J. L. H. van Leenders

**Affiliations:** 1 Anser Prostate Operation Clinic, Maasstadweg 21, 3079 DZ Rotterdam, The Netherlands; 2 Department of Pathology, Maasstad Hospital, Rotterdam, The Netherlands; 3 Department of Urology, Maasstad Hospital, Rotterdam, The Netherlands; 4 Department of Pathology, Erasmus MC Cancer Institute, University Medical Centre, Rotterdam, The Netherlands; 5 Department of Radiology, Erasmus MC Cancer Institute, University Medical Centre, Rotterdam, The Netherlands; 6 Department of Urology, Erasmus MC Cancer Institute, University Medical Centre, Rotterdam, The Netherlands; 7 Department of Urology, Franciscus Gasthuis & Vlietland, Rotterdam, The Netherlands

**Keywords:** Prostate cancer, Gleason score, Cribriform, Percentage

## Abstract

**Purpose:**

To identify parameters to predict upgrading in biopsy Grade Group (GG) 2 prostate cancer patients without cribriform and intraductal carcinoma (CR/IDC) on biopsy.

**Methods:**

Preoperative biopsies from 657 men undergoing radical prostatectomy (RP) for prostate cancer were reviewed for GG, presence of CR/IDC, percentage Gleason pattern 4, and tumor length. In men with biopsy GG2 without CR/IDC (*n* = 196), clinicopathologic features were compared between those with GG1 or GG2 without CR/IDC on RP (GG ≤ 2−) and those with GG2 with CR/IDC or any GG > 2 (GG ≥ 2+). Logistic regression analysis was used to predict upgrading in the biopsy cohort.

**Results:**

In total 283 men had biopsy GG2 of whom 87 (30.7%) had CR/IDC and 196 (69.3%) did not. CR/IDC status in matched biopsy and RP specimens was concordant in 179 (63.3%) and discordant in 79 (27.9%) cases (sensitivity 45.1%; specificity 92.6%). Of 196 biopsy GG2 men without CR/IDC, 106 (54.1%) had GG ≥ 2+ on RP. Multivariable logistic regression analysis showed that age [odds ratio (OR): 1.85, 95% confidence interval (CI)1.09–3.20; *p* = 0.025], percentage Gleason pattern 4 (OR 1.54, 95% CI 1.17–2.07; *p* = 0.003), PI-RADS 5 lesion (OR 2.17, 95% CI 1.03–4.70; *p* = 0.045) and clinical stage T3 (OR 3.60; 95% CI 1.08–14.50; *p* = 0.049) were independent parameters to predict upgrading to GG ≥  2+ on RP in these men.

**Conclusions:**

Age, clinical stage T3, percentage Gleason pattern 4 and presence of PI-RADS 5 lesions are independent predictors for upgrading in men with biopsy GG2 without CR/IDC. These findings allow for improved clinical decision-making on surveillance eligibility in intermediate-risk prostate cancer patients.

## Introduction

Grade Group (GG) and invasive cribriform and/or intraductal carcinoma (CR/IDC) are important pathological parameters for clinical decision-making in prostate cancer patients. Active surveillance is offered to low-risk patients and increasingly to men with favorable intermediate-risk GG2 disease [1]. Patients with GG1 and GG2 without CR/IDC both have very low risk of developing metastases and dying from prostate cancer, while outcome of GG2 men with CR/IDC is significantly worse [2–4]. According to the EAU guidelines, therefore, biopsy GG2 patients with CR/IDC should be excluded from active surveillance [5].

It is well known that prostate cancer grade at biopsy poorly reflects the actual tumor grade in the entire prostate due to sampling constraints. Thus, up- and downgrading of GG at radical prostatectomy (RP) are frequently observed. Biopsy GG1 is upgraded in 43–67%, while GG2 is upgraded in 19–23% and downgraded in 7% of patients, respectively [6–8]. Upgrading is less likely with multiparametric magnetic resonance imaging (mpMRI)-targeted biopsies, while downgrading is not affected [9]. Likewise, three studies have demonstrated significant sampling artifact for CR/IDC in matched biopsy and RP specimens [10–12]. Overall sensitivity for detecting CR/IDC at biopsy is 43–57% with 87–97% specificity, indicating that about half of CR/IDC lesions present in the prostate are missed on biopsy. More specifically, men with biopsy GG2 without cribriform pattern do have CR/IDC at subsequent RP in 40% of cases [10]. Since GG2 men without CR/IDC may currently be eligible for active surveillance, under-sampling of cribriform pattern might lead to undertreatment of patients with potentially more aggressive disease.

For optimal management of biopsy GG2 men without CR/IDC, it is important to distinguish those with unsampled cribriform pattern or ≥ GG3 disease from the ones with favorable disease who might be eligible to surveillance. Various clinical, radiological and pathological parameters may result in better risk stratification of biopsy GG2 prostate cancer patients. The aim of this study was to identify predictive parameters for tumor upgrading of GG2 prostate cancer patients without cribriform pattern at biopsy.

## Materials and methods

### Study population

The Anser Prostate Network is a collaboration of eight medical centers in the Netherlands, which refer all patients scheduled for RP to one high-volume Anser Prostate operation Clinic, located in Maasstad Hospital, Rotterdam, the Netherlands. Diagnostic biopsies, imaging, and multidisciplinary team meetings are performed in the referring centers. In this study, men undergoing RP for prostate cancer in the Anser Prostate operation Clinic between September 2018 and April 2020 were included. If patients had received mpMRI, the Prostate Imaging Reporting and Data System (PI-RADS) score was reported by the radiologist of the referring centers, and targeted biopsies had been taken for all PI-RADS ≥ 3 lesions. The study was approved by the institutional Medical Ethical Committee (METC-2019-0352).

### Pathological evaluation

All RPs were formalin-fixed, transversely sectioned into 4-mm slices from apex to base, completely embedded and stained with hematoxylin and eosin. The RP slides were evaluated by five pathologists with an interest in genitourinary pathology. The Gleason score/GG according to the 2014 ISUP/ 2016 WHO recommendations, presence of CR/IDC, pT-stage, and surgical margin status were recorded for each patient [13, 14]. No distinction was made between invasive cribriform and intraductal carcinoma. Biopsies prior to RP were collected and reviewed by one genitourinary pathologist (GvL) for research purposes. Patients of whom not all slides were available for pathological revision were excluded. At biopsy revision, GG, cumulative tumor length, CR/IDC, and percentage Gleason pattern 4 were assessed for each side and target lesion separately, according to the 2014 International Society of Urological Pathology (ISUP) recommendations [13]. In this study, we used the GG and percentage Gleason pattern 4 from the side with the worst Gleason score for comparison with subsequent RP outcome, while cribriform status was coalesced from all biopsy sides.

### Statistical analysis

Categorical parameters of biopsy GGs were compared with chi-square test and continuous variables with Mann–Whitney test. Logistic regression analysis was used to predict upgrading in biopsy GG2 without CR/IDC where RP outcome was classified into two groups: GG1 or GG2 without CR/IDC (GG2−), and GG3 to 5 or GG2 with cribriform pattern (GG ≥ 2+). Statistical analyses were performed using R version 3.6.2. A *p* value of ≤ 0.05 was considered statistically significant.

## Results

### Patient characteristics

In total 657 patients with matched RP and reviewed biopsies were identified, of whom 105 (16%) had biopsy GG1, 283 (43.1%) GG2, 189 (28.8%) GG3, 35 (5.3%) GG4 and 45 (6.8%) GG5. The median age of the biopsy GG2 patients (*n* = 283) at the time of operation was 67 years (interquartile range (IQR) 63–71), and their median preoperative PSA level was 8.4 ng/ml (IQR 6.2–12.0) (Table 1). Eighty-three (29.3%) patients had undergone systematic and target biopsies, 144 (50.9%) systematic biopsies only, and 56 (19.8%) target biopsies only. The incidence of CR/IDC at biopsy was 87/283 (30.7%) and at RP 175 (61.8%); no cribriform pattern was identified in biopsies of 196 (69.3%) men. The clinicopathological features of biopsy GG2 men with and without CR/IDC are shown in Table 1. Men with biopsy CR/IDC had higher percentage of positive biopsies (*p* < 0.001), higher cumulative tumor length (*p* < 0.001), higher percentage Gleason pattern 4 (*p* < 0.001), and higher Grade Group on RP (*p* = 0.004). pT-stage and surgical margin status were not significantly different between both biopsy cohorts.

**Table 1:** Pre- and postoperative patient characteristics of men with biopsy GG2

Parameters	All patients	GG2-	GG2+	*p* value
Preoperative characteristics	*N* = 283	*N* = 196	*N* = 87	
Age (years), median (IQR)	67.0 (63–71.0)	67.0 (62.8–71.0)	68 (63–71)	0.999
PSA (ng/ml), median (IQR)	8.4 (6.2–12.0)	8.9 (6.5–12.2)	7.4 (5.7–11.9)	0.152
Total biopsy number, median (IQR)	10 (8–12)	10 (8–12)	10 (8–12)	0.581
Percentage of PCa-positive biopsies, median (IQR)	50.0% (37.5–76.0%)	50.0% (33.3–75.0%)	62.5 (45.0–86.1%)	< 0.001
Cumulative tumor length in mm, median (IQR)	20.5 (12.3–37.2)	18.0 (10.3–31.0)	31.0 (17.5–47.3)	< 0.001
Percentage of Gleason pattern 4, median (IQR)	20.0% (10.0–30.0%	16.0% (10.0–30.0%)	30.0% (20.0–40.0%)	< 0.001
CR/IDC on biopsy, *n* (%)	87 (30.7%)			
Biopsy, *n* (%)				0.799
Systematic and target biopsies	83 (29.3%)	57 (29.1%)	26 (29.9%)	
Only systematic biopsies	144 (50.9%)	102 (52.0%)	42 (48.3%)	
Only target biopsies	56 (19.8%)	37 (18.9%)	19 (21.8%)	
Postoperative characteristics				
Grade Group RP, *n* (%)				0.004
1	11 (3.9%)	11 (5.6%)	0 (0%)	
2	204 (72.1%)	148 (75.5%)	56 (64.4%)	
3	58 (20.5%)	31 (15.8%)	27 (31.0%)	
4	7 (2.5%)	4 (2.0%)	3 (3.4%)	
5	3 (1.1%)	2 (1.0%)	1 (1.1%)	
Pathological stage, *n* (%)				0.927
pT2	169 (59.7%)	117 (59.7%)	52 (59.8%)	
pT3a	81 (28.6%)	57 (29.1%)	24 (27.6%)	
pT3b	33 (11.7%)	22 (11.2%)	11 (12.6%)	
CR/IDC on RP, *n* (%)	175 (61.8%)	96 (49.0%)	79 (90.8%)	< 0.001
Positive surgical margin, *n* (%)	79 (27.9%)	58 (29.6%)	21 (24.1%)	0.424

### Grade Group 2 and CR/IDC concordance

Biopsy and RP tumor grade were concordant in 204/283 (72.1%) GG2 men, with upgrading in 68 (24%) and downgrading in 11 (3.9%). On RP, 11 (3.9%) men had GG1, 204 (72.1%) GG2, 58 (20.5%) GG3, and 10 (3.6%) GG4 or 5. CR/IDC was concordant in 179 (63.3%) matched biopsy and RP specimens; in 79 (27.9%) CR/IDC was present and in 100 (35.3%) it was absent in both. In 96 (33.9%) cases no CR/IDC was observed in the biopsy, but it was present in the RP specimen. CR/IDC was observed in the biopsy but not in matched RP in 8 (2.8%) men. The sensitivity of biopsies to detect CR/IDC was 45.1% and specificity was 92.6%.

### Upgrading in men with biopsy 2 without CR/IDC

In total 186 out of 283 (65.7%) men with biopsy GG2 had GG2 with CR/IDC or GG3 to 5 regardless of cribriform pattern on RP. When stratified for the presence of CR/IDC on biopsy, 106/196 (54.1%) biopsy GG2 men without CR/IDC had GG ≥  2+ on RP as compared to 80/87 (92.0%) of men with cribriform pattern on biopsy. Table 2 summarizes the pre-operative characteristics of biopsy GG2 men without CR/IDC, who were upgraded to GG ≥ 2+ (* n* = 106) compared to those with GG1 or GG2 without cribriform pattern (*n* = 90) at matched RP. Biopsy percentage pattern 4 (*p* = 0.008) and clinical stage (*p* = 0.011) were significantly higher in men who were upgraded at RP. Whereas age (*p* = 0.077), PSA density (*p* = 0.055) and PI-RADS score (*p* = 0.075) also tended to be higher in those men, these did not reach conventional measure of significance. No significant difference was found in number of biopsies, percentage of positive biopsies, or cumulative tumor length.

**Table 2:** Upgrading to GG ≥ 2+ on RP in men with biopsy GG2 without CR/IDC

GG2−	GG1 or GG2 CRIDC− *N* = 90	GG2 CRIDC+ or GG > 2 *N* = 106	*p* value
Age, median (IQR)	66.0 (60.0–70.0)	67.5 (64.0–71.0)	0.077
PSA, median (IQR)	8.2 (6.1–11.8)	9.5 (6.6–12.3)	0.190
Clinical Stage, *n* (%)			0.011
T1	51 (56.7%)	50 (47.2%)	
T2a	22 (24.4%)	21 (19.8%)	
T2b	5 (5.6%)	11 (10.4%)	
T2c	8 (8.9%)	4 (3.8%)	
T3	4 (4.4%)	20 (18.9%)	
Percentage of Gleason pattern 4, *n* (%)			0.008
0–9%	20 (22.2%)	12 (11.3%)	
10–19%	38 (42.2%)	29 (27.4%)	
20–29%	13 (14.4%)	26 (24.5%)	
30–39%	11 (12.2%)	27 (25.5%)	
40–50%	8 (8.9%)	12 (11.3%)	
MRI PI-RADS, *n* (%)			0.075
No MRI	14 (15.6%)	17 (16.0%)	
Not suspected	6 (6.7%)	3 (2.8%)	
3	6 (6.7%)	7 (6.6%)	
4	41 (45.6%)	35 (33.0%)	
5	20 (22.2%)	43 (40.6%)	
Unknown	3 (3.3%)	1 (0.9%)	
Number of biopsies, median (IQR)	10 (8–12)	10 (8–12)	0.884
Percentage of PCa-positive biopsies, median (IQR)	50.0% (35.7–66.7%)	50.0% (33.3–75.0%)	0.911
Percentage of PCa-positive biopsies (target counting as one), median (IQR)	50.0% (30.9–74.4%)	50.0% (31.4–80.0%)	0.989
Percentage of PCa-positive biopsies (according to PRIAS), *n* (%)			1
≤ 50%	51 (56.7%)	59 (55.7%)	
> 50%	39 (43.3%)	47 (44.3%)	
Cumulative tumor length, median (IQR)	16.7 (9.6–29.4)	19.7 (10.4–34.8)	0.232
Biopsy, *n* (%)			0.145
Systematic and target biopsies	30 (33.3%)	27 (25.5%)	
Only systematic biopsies	40 (44.4%)	62 (58.5%)	
Only target biopsies	20 (22.2%)	17 (16.0%)	

### Prediction of GG ≥ 2+ in cribriform-negative GG2 biopsies

Logistic regression showed that age, clinical stage T3, percentage Gleason pattern 4, and MRI PI-RADS 5 lesions were all associated with GG ≥ 2+ on subsequent RP in univariate analysis, whereas PSA, number of biopsies, percentage of cancer-positive biopsies and cumulative tumor length were not (Table 3). In multivariable analysis, age (odds ratio (OR): 1.85, 95% confidence interval (CI): 1.09–3.20; *p* = 0.025), percentage Gleason pattern 4 (OR 1.54, 95% CI 1.17–2.07; *p* = 0.003), PI-RADS 5 lesions (OR 2.17, 95% CI 1.03–4.70; *p* = 0.045), and clinical stage T3 (OR 3.60; 95% CI 1.08–14.50; *p* = 0.049) remained significant for predicting RP upgrading in biopsy GG2 men without cribriform pattern.

**Table 3:** Uni- and multivariable logistic regression to predict GG2 with CR/IDC or higher on RP

Logistic regression	Biopsy GG2-				
	Univariable			Multivariable	
	GG1 or GG2- (RP)	OR (95% CI)		GG2+ or GG > 2 (RP)	*p* value
	Ref			OR (95% CI)	
Age (per 10 years)		1.79 (1.11–2.92)	0.018	1.85 (1.09–3.20)	0.025
PSA(log2)		1.20 (0.85–1.71)	0.315		
Clinical Stage					
T1	Ref				
T2		1.05 (0.57–1.93)	0.877	1.04 (0.54–2.03)	0.904
T3		5.10 (1.78–18.48)	0.005	3.60 (1.08–14.50)	0.049
MRI PI-RADS					
< 5	Ref				
5		2.53 (1.32–4.98)	0.006	2.17 (1.03–4.70)	0.045
No MRI		1.43 (0.64–3.26)	0.387	1.04 (0.42–2.58)	0.931
Percentage of Gleason pattern 4 on biopsy (per 10%)		1.50 (1.16–1.98)	0.003	1.54 (1.17–2.07)	0.003
Number of biopsies		1.01 (0.94–1.08)	0.873		
Percentage of PCa-positive biopsies		1.00 (0.99–1.01)	0.892		
Cumulative tumor length on biopsy (mm)		1.01 (1.00–1.03)	0.242		
Presence of targeted biopsies		0.57 (0.32–1.00)	0.051	0.61 (0.31–1.18)	0.143

## Discussion

Men with biopsy GG2 prostate cancer with CR/IDC are ineligible for active surveillance, because the presence of CR/IDC is associated with increased risk of metastases and biochemical recurrence [4, 5, 15]. On the other hand, active surveillance could be a safe option for GG2 men without CR/IDC. Biopsy sampling constraints might, however, lead to inclusion of men with more aggressive tumor features such as GG3 or CR/IDC. This and other studies have shown that CR/IDC is missed in about half of biopsy GG2 patients, suggesting these men are at risk of being denied active treatment [10, 12]. Identification of those men at risk for being undergraded at biopsy is crucial for individual clinical management. In the current study of 196 biopsy GG2 men without CR/IDC, we show that 54.1% had GG3 or higher and/or GG2 with CR/IDC on subsequent RP. Higher age, cT3 stage, increasing percentage Gleason pattern 4 and presence of mpMRI PI-RADS 5 lesions were independent factors for tumor upgrading, while PSA and biopsy tumor volume did not have added value. These results allow for better risk stratification and clinical decision-making in men with biopsy GG2.

Three groups have investigated the concordance of CR/IDC on biopsy and RP [10–12]. In our study, we found low sensitivity of 45.1% and high specificity of 92.6% for presence of CR/IDC in biopsy GG2 patients. This is in the range of 43–57% overall sensitivity for CR/IDC in all biopsy GG and 38–48% sensitivity in biopsy GG2 reported by others [10–12]. Sampling constraints are the most important reason for this low sensitivity in all studies. The high specificity of 92.6% in this study is also in agreement with 87–100% specificity range from the other groups [10–12]. Variability between studies might result from differences in cohort composition and inter-observer variability. For instance, in the concordance study of Hollemans et al. all biopsies and RP specimens were scored by two pathologists in common sessions [10]. In the current study, all biopsies were reviewed by one pathologist but the RP specimens were evaluated by an independent group of five pathologists. Apart from inter-observer agreement for diagnosing CR/IDC, even in entirely embedded RP specimens only < 1% of the tissue, i.e. a 5 μm HE slice from 3–4 mm thick tissue slide, is microscopically evaluated and could lead to missing occult cribriform lesions in resection specimens. This might explain that in some cases with cribriform pattern on biopsy, no CR/IDC was identified at subsequent RP.

Active surveillance is increasingly being offered to patients with favorable intermediate risk GG2 prostate cancer patients [16, 17]. There is no global consensus on surveillance eligibility criteria and practices use different inclusion criteria [1].The EAU and National Comprehensive Cancer Network (NCCN) both recommend active surveillance for selected patients with favorable GG2 with PSA < 10 ng/ml and ≤ cT2a disease [5, 18]. Furthermore, NCCN takes the number of positive cores into account, while the EAU guideline includes men with < 10% Gleason pattern 4 and without CR/IDC. Although some have indicated that presence of PI-RADS 5 lesion on MRI is associated with more aggressive disease, this is not included as a criterium by EAU or NCCN yet [19–21]. In the current study, clinical stage T3, presence of mpMRI PI-RADS 5 lesions and increasing biopsy percentage Gleason pattern 4 were all significant predictors for upgrading to GG ≥ 2 on RP, while biopsy tumor volume and PSA did not have independent value. The potential significance of PI-RADS 5 lesions in biopsy GG2 men was also found by others [10, 19–21]. Cribriform architecture by definition lacks intervening stroma resulting in high cell density, and is associated with high tumor volume. We postulate that both pathological characteristics are the reason that cribriform lesions are more frequently visible as MRI PI-RADS 5 lesions.

The inclusion of percentage Gleason pattern 4 as surveillance eligibility criterion was initially prompted by pathological inter-observer variability in recognizing and interpreting small pattern 4 structures [22]. Our group has shown that percentage Gleason pattern 4 and presence of CR/IDC are related on both biopsy and RP specimens [23, 24]. In a GG2 biopsy cohort of 370 men, Kweldam et al. found that those with < 10% Gleason pattern 4 had CR/IDC in 6%, while this increased to 44% in biopsies with 25–50% pattern 4 [23]. In the current study, we found that incremental percentage pattern 4 was significantly associated with more aggressive disease at RP. It is however difficult to recommend a specific cut-off for surveillance eligibility. While 10% is regularly applied in clinical practice, patients with higher percentages should not be denied active surveillance by definition, particularly if other favorable factors are present. Of interest, we did not find the number of positive biopsies or tumor volume to be associated with adverse outcome in our model. This might be explained by variability of biopsy templates in clinical practice including systematic, targeted or both types of biopsies, resulting in significant heterogeneity of percentage positive biopsies and tumor volume assessment. Finally, apart from above-mentioned clinically relevant parameters, patients’ personal attitude to the pros and cons of active surveillance versus treatment are most important.

The strengths of this study are the relatively large number of men with matched biopsy and operation specimen who were all operated in one high-volume center and detailed central pathological biopsy review. Albeit the RP specimens were not centrally reviewed, inter-observer agreement was optimized by common educational sessions [25]. A disadvantage is the heterogeneity of the study population with variability in biopsy procedures and MRI assessment, which however reflects current daily practice. Finally, whereas the current study provides evidence for relevant factors to be considered for active surveillance in biopsy GG2 men, translation towards a comprehensive clinical decision-tree or risk calculator is still required for practical implementation. The number of patients in this study was, however, still too low to validate such a risk-calculator.

In conclusion, 54.1% of biopsy GG2 men without CR/IDC had more aggressive ≥ GG3 or GG2 with CR/IDC prostate cancer at subsequent RP. Age, clinical stage T3, percentage Gleason pattern 4, and presence of mpMRI PI-RADS 5 lesions were all independent predictors for upgrading men with biopsy GG2 without CR/IDC. These findings facilitate improved clinical decision-making on surveillance eligibility in intermediate-risk prostate cancer patients.
